# Child Survival and Annual Crop Yield Reductions in Rural Burkina Faso: Critical Windows of Vulnerability Around Early-Life Development

**DOI:** 10.1093/aje/kwad068

**Published:** 2023-04-28

**Authors:** Kristine Belesova, Antonio Gasparrini, Paul Wilkinson, Ali Sié, Rainer Sauerborn

**Keywords:** agriculture, child health, child mortality, climate change, crop yield, early-life development, survival, vulnerability

## Abstract

Populations that are reliant on subsistence farming are particularly vulnerable to climatic effects on crop yields. However, empirical evidence on the role of the timing of exposure to crop yield deficits in early-life development is limited. We examined the relationship between child survival and annual crop yield reductions at different stages of early-life development in a subsistence farming population in Burkina Faso. Using shared frailty Cox proportional hazards models adjusting for confounders, we analyzed 57,288 children under 5 years of age followed by the Nouna Health and Demographic Surveillance System (1994–2016) in relation to provincial food-crop yield levels experienced in 5 nonoverlapping time windows (12 months before conception, gestation, birth–age 5.9 months, ages 6.0 months–1.9 years, and ages 2.0–4.9 years) and their aggregates (birth–1.9 years, first 1,000 days from conception, and birth–4.9 years). Of the nonoverlapping windows, point estimates were largest for child survival related to food-crop yields for the time window of 6.0 months–1.9 years: The adjusted mortality hazard ratio was 1.10 (95% confidence interval: 1.03, 1.19) for a 90th-to-10th percentile yield reduction. These findings suggest that child survival in this setting is particularly vulnerable to cereal-crop yield reductions during the period of nonexclusive breastfeeding.

## Abbreviations

CI: confidence interval
FCPI: food crop productivity index
HDSS: Health and Demographic Surveillance System
HR: hazard ratio
NDVI: normalized difference vegetation index

Children in the subsistence farming populations of sub-Saharan Africa are often exposed to food insecurity and may experience heightened risk of undernutrition and illness when crop yields are low (1). Climate change may aggravate these risks as changing temperature and rainfall patterns lead to greater fluctuations and a less predictable food supply, particularly for households that are reliant on rain-fed agriculture (2, 3).

Adequate food intake in early life is essential for children’s cognitive and physical development and lifelong health. The 1,000 days from conception to 2 years of age are particularly critical due to high nutritional requirements for rapid growth and neurodevelopment (4–7). Inadequate energy and nutrient intake during this period is likely to result in stunting, lifelong deficits in brain function, and increased likelihood of subsequent cardiovascular, endocrine, and metabolic diseases, including obesity and impaired kidney function, as well as mental illness (4, 7, 8). Similarly, maternal food intake before conception can influence the offspring’s risk of preterm birth and immune system and brain development (9, 10). The impacts of food intake restriction before conception and in early life are not always reflected by changes in children’s anthropometric characteristics, as those can operate independently through early-life nutritional programming of the immune system at the epigenetic level (11–13).

Establishing the time frames of greatest vulnerability to variations in crop yields can help identify opportunities for improving nutritional practices and programs. Yet, evidence on effects of exposure to crop yield reductions (and such proxies as weather patterns during the growing season) at different stages of early life in vulnerable subsistence farming populations is scarce and inconsistent (1, 14). A limited number of researchers have studied associations of child growth with the normalized difference vegetation index (NDVI) and exposure to rainfall shocks during the crop-growing season 1 year before birth versus the first year of a child’s life (15, 16), and some have also studied associations with exposures in the second year of life (17, 18). Their results were mixed, with varying evidence for associations during different exposure windows in different settings. A more systematic examination is needed. Because annual crop yield deficits are projected to worsen for West Africa under projected climate change (19), better evidence is especially important to inform health- and nutrition-sensitive adaptation efforts to protect children.

In previous work carried out in Burkina Faso, we showed that child survival to 4.9 years of age was associated with crop yield levels in the child’s year of birth, without distinguishing the effect of in utero exposures from those incurred during the first year of life (20). In the current study, we examined child survival in relation to exposure to crop yield fluctuations in 5 time periods prior to birth and in early childhood: 1) maternal exposure before conception, 2) exposures experienced in utero, and exposures experienced 3) in the first 5.9 months after birth, 4) from 6.0 months of age to 1.9 years of age, and 5) from 2 years of age to 4.9 years of age, as well as their aggregates: lifetime average exposure, exposure during the 1,000 days from conception, and exposure at ≤1.9 years of age. We used the latest version of a longitudinal data set comprising 24 years of follow-up from the Nouna Health and Demographic Surveillance System (HDSS). It was larger than the data set used in our earlier work, enhancing the statistical power of the current analyses (20).

## METHODS

### Study population

Our study population, which has been surveyed as an open dynamic continuous cohort as a part of the Nouna HDSS by the Centre de Recherche en Santé de Nouna, is located in the Kossi Province of Burkina Faso (21). The population under surveillance has grown from 26,626 in 1992 to 150,000 currently (2022) through natural population growth and incorporation of additional villages (21). It relies almost entirely on rain-fed subsistence agriculture with 1 agricultural season per annum, with food being harvested around September.

Our data were obtained as follows.

#### Outcome: child mortality/survival data.

We acquired the latest available Nouna HDSS data covering 57,288 children under 5 years of age who were followed up over the period 1994–2016, including vital and migration events recorded through surveys that took place every 3 months until 2006 and every 4 months thereafter. Additionally, a control census was undertaken every 5 years. Individuals born before 1994, those born outside of the Nouna HDSS area, and those with missing records on their month of birth, death, or migration were excluded. Data for the years 1992–1993 were not used for analyses because of concerns over data incompleteness while the surveillance system was under development.

#### Exposure: agricultural yield data.

We obtained data on crop yields (kg/ha) in Kossi Province during 1994–2016 from the Annual Agricultural Survey of Burkina Faso (Agricultural Statistics Service of Burkina Faso) (22) and calculated the food crop productivity index (FCPI) using previously published methods (20). The FCPI represents a weighted average of the yield of the 5 main food crops in Kossi Province (millet, sorghum, maize, fonio, and rice) relative to their annual mean yield during the period 1994–2016, expressed as a percentage of the period average. The amount of each type of crop was weighted by the proportion of the harvest that it contributed to the total harvest across the 5 crop types grown each year in the province.

#### Covariates: sociodemographic data.

HDSS data included information on individual demographic and socioeconomic characteristics (sex, ethnicity, religion, mother’s and father’s ability to read, familial relationships, rural vs. semirural residence) and household characteristics (the presence of any household members in occupations outside of agriculture and a wealth index developed by Schoeps et al. (23), coded to quartiles). The wealth index represents household housing conditions (e.g., dwelling type, type of roof and walls, water source in the dry and rainy seasons, type of toilet and sanitation, source of lighting, energy source for cooking) and asset ownership (e.g., means of transportation, agricultural assets, and ownership of household items such as a radio, television, refrigerator, or modern stove) based on the data for 2009 (23). Because the wealth index data were available only for 2009, we assigned the index value from 2009 to all years through which the household could be traced in our data set over the course of the analysis period from 1994 to 2016. We reclassified any missing data on individual and socioeconomic characteristics into a separate category labeled “unclassified.” We also acquired data on infrastructural characteristics of Nouna HDSS villages (presence of drilled water wells, markets, health-care facilities, and the quality of road connections) from a geographic information system database of the Centre de Recherche en Santé de Nouna, from which we used principal component analysis to construct a village infrastructural development index (quartiles).

### Analyses

**Figure 1:**
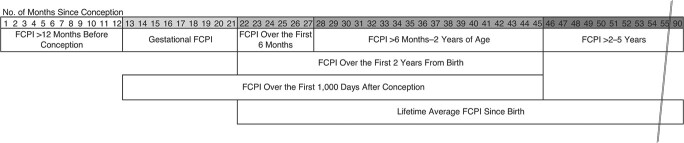
Timing of exposure to reduced crop yields in a study of the relationship between child survival and crop yield variations at different stages of child development, Nouna Health and Demographic Surveillance System, Burkina Faso, 1994–2016. FCPI, food crop productivity index.

To examine child survival in relation to exposure to crop yield variations at different stages of child development, we calculated exposure indices for the following periods (Figure 1):

1. the 12 months before conception, a window representing the mother’s food intake that can affect the offspring’s development and health (9, 10);
2. the first 1,000 days from conception (approximated as 270 days before birth and 730 days after birth), which we further separated into 2 subperiods:
  - in utero (approximated as 270 days before birth) and
  - the first 2 years after birth, further segmented into the subperiods corresponding to different breastfeeding regimen recommendations issued by the World Health Organization (24):
    i. birth to age 5.9 months—the period of exclusive breastfeeding, and
    ii. ages 6.0 months to 1.9 years—the period of nonexclusive breastfeeding;
3. iii. ages 2.0–4.9 years; and
4. iv. birth to age 4.9 years.

These exposure indices were calculated using data on each child’s date of birth and exit from surveillance in relation to the timing of each agricultural harvest, assumed to start on September 1. The index for each time window was calculated as a weighted average of the FCPI levels during the agricultural years in which the period fell as the child aged—that is, from the start of the window to death or exit from observation or the end of the window, weighted by the proportion of time falling in each agricultural year. For example, if a child dies at the age of 1 year, his lifetime average exposure reflects the FCPI levels experienced in this 1 year of life; if a child dies at the age of 3 years, his lifetime average exposure represents the weighted average of FCPI levels experienced throughout the first 3 years of life. For comparability with our earlier work (20), we replicated the survival analyses in relation to FCPI exposure in the agricultural year of birth, using the updated data set. The year-of-birth exposure only considered the FCPI of the harvest year in which the child was born, disregarding the exposure to other FCPI levels in earlier and later agricultural years.

We examined associations of each exposure index with survival using tabulations, Kaplan-Meier plots, and Cox proportional hazards models with shared frailty specified by village and age used as the analysis time. First, a separate model was fitted for each exposure index separately. We then fitted models that simultaneously included indicators for more than 1 exposure window. Child observations that were lost to follow-up before the child reached the age of 5 years were censored on the date of exit from surveillance. Models with the exposure of FCPI over 2.0–4.9 years of age were based only on observations of children who survived and were present in the HDSS at the age of 2 years.

For Kaplan-Meier plots, each exposure index was transformed into a binary indicator above the period average FCPI versus below the period average FCPI. For the Cox models, we used continuous exposure indices. The survival hazard ratios (HRs) are reported in relation to the change in exposure from the 90th percentile of exposure to the 10th percentile.

All Cox models adjusted for different combinations of potential confounders, which we determined a priori (1, 16, 25, 26): 1) random effects at the village level, with subsequent addition of adjustments for 2) the establishment of a local undernutrition treatment program (indicator of a step change in 2007) and a linear time trend (i.e., year fitted as a linear term to control for any long- term, continuous changes in child mortality and crop yields), and 3) all time-invariant sociodemographic characteristics of the children, their households, and their villages: season of birth, sex, ethnicity, religion, mother’s and father’s ability to read, household’s wealth index, the presence of any household members involved in a nonagricultural occupation, level of village infrastructural development, and semirural versus rural residence.

To examine whether expansion of the Nouna HDSS population through the addition of new villages in the years 2000 and 2004 could have biased our analyses, we performed sensitivity analyses by restricting the data set to only those villages that had been part of the HDSS since its inception.

We stratified the analyses of child survival in relation to one of the FCPI exposure indices by wealth index categories to explore whether and how the association varied with household wealth.

Statistical analyses were performed using Stata 16.1 (StataCorp LLC, College Station, Texas) (27).

The study was conducted following the ethical standards of the Declaration of Helsinki (28) and was approved by the Ethics Committee of the Medical Faculty of Heidelberg University (Heidelberg, Germany) and the Comité Institutionnel d’Ethique du Centre de Recherche en Santé de Nouna (Nouna, Kossi Province, Burkina Faso). Informed consent was obtained by the Centre de Recherche en Santé de Nouna from all subjects at the time of health and demographic data collection.

## RESULTS

The characteristics of the study population are presented in Table 1. There were 5,331 deaths, with an average mortality rate of 26.22 deaths per 1,000 person-years.

**Table 1:** Characteristics of Children Included in the Nouna Health and Demographic Surveillance System, Burkina Faso, 1994–2016

**Factor**	**No. of** **Children**	**% of** **Children**	**No. of** **Deaths**	**P-Y at** **Risk**	**Mortality Rate** **per 1,000 P-Y**
Age group, years^a^					
<1	57,288	100	2,440	6,462	377.58
≥1	48,828	85	1,527	12,705	120.19
≥2	41,460	72	902	16,593	54.36
≥3	35,240	62	313	17,004	18.41
≥4	30,498	53	149	150,529	0.99
Sex					
Male	28,793	50	2,799	101,963	27.45
Female	28,495	50	2,532	101,330	24.99
Ethnicity					
Bwamu	14,501	25	1,221	52,126	23.42
Dafing	22,426	39	2,345	79,663	29.44
Mossi	10,014	17	784	35,820	21.89
Peul/Fulani	5,760	10	643	19,686	32.66
Samo	3,435	6	258	12,254	21.05
Other	1,081	2	74	3,519	21.03
Unclassified	71	0	6	224	26.81
Religion					
Animist	2,700	5	335	9,872	33.93
Catholic	15,187	27	1,191	54,421	21.88
Muslim	36,696	64	3,624	129,186	28.05
Protestant	2,552	4	168	9,294	18.08
Other	107	0	5	382	13.08
Unclassified	46	0	8	137	58.59
Mother’s ability to read					
Unable	29,828	52	3,264	127,820	25.54
With difficulty	1,680	3	128	7,280	17.58
Easily	1,746	3	105	7,238	14.51
Unclassified	24,034	42	1,834	60,955	30.09
Father’s ability to read					
Unable	26,828	47	2,933	114,066	25.71
With difficulty	3,667	6	321	15,858	20.24
Easily	3,331	6	231	14,231	16.23
Unclassified	23,462	41	1,846	59,137	31.22
Season at birth					
September–November	15,624	27	1,576	55,662	28.31
December–February	13,287	23	1,289	47,348	27.22
March–May	14,257	25	1,222	50,608	24.15
June–August	14,120	25	1,244	49,675	25.04
≥1 household member occupied outside agriculture^b^					
No	41,947	73	3,727	146,895	25.37
Yes	4,110	7	416	15,289	27.21
Unclassified	11,231	20	1,188	41,109	28.90
Wealth					
Level 1 (poorest)	10,129	18	1,133	37,846	29.94
Level 2	10,772	19	1,143	39,613	28.85
Level 3	11,674	20	1,082	43,039	25.14
Level 4 (wealthiest)	9,264	16	775	34,101	22.73
Unclassified	15,449	27	1,198	48,694	24.60
Village infrastructure level					
Level 1 (lowest)	18,369	32	2,004	64,514	31.06
Level 2	18,700	33	1,761	66,332	26.55
Level 3	7,041	12	793	25,974	30.53
Level 4 (highest)	13,178	23	773	46,473	16.63

Abbreviation: P-Y, person-years.

^a^ The number of children in each of the subsequent age groups is a subset of the survivors from the preceding age group.

^b^ In addition to the agricultural work of other household members.

Across all of the exposure windows, FCPI values ranged from 49% to 139% of the period average weighted provincial yield. Table 2 gives the statistics by exposure window. Most of these indicators were largely uncorrelated (see Web Table 1, available at https://doi.org/10.1093/aje/kwad068). The variation in crop-specific yield and FCPI values across the study years is illustrated in Web Figure 1.

**Table 2:** Food Crop Productivity Index Values for Exposure to Reduced Food-Crop Yields, According to Timing of Exposure, Among Mothers and Children in the Nouna Health and Demographic Surveillance System, Burkina Faso, 1993–2016

**FCPI Exposure Period**	**Median,** **%** ^**a**^	**Range,** **%** ^**a**^	**10th and 90th** **Percentiles** ^**b**^	**90th–10th** **Percentile Range** ^**c**^
Before conception	100	52–138	78, 119	−41
First 1,000 days from conception	105	49–139	80, 116	−36
Gestation	101	49–139	78, 122	−44
Age ≤1.9 years	103	61–134	80, 118	−38
Age ≤5.9 months	102	61–134	80, 120	−40
Ages 6.0 months–1.9 years	104	61–134	81, 115	−34
Ages 2.0–4.9 years	105	61–134	80, 119	−39
Child’s lifetime average	105	61–134	81, 115	−34
First year after birth	100	61–134	78, 120	−42

Abbreviation: FCPI, food crop productivity index.

^a^ Percentage of the period average weighted provincial yield.

^b^ Values of the 10th and 90th percentiles of each exposure indicator.

^c^ Difference between the 10th and 90th percentile values of each exposure indicator.

**Figure 2:**
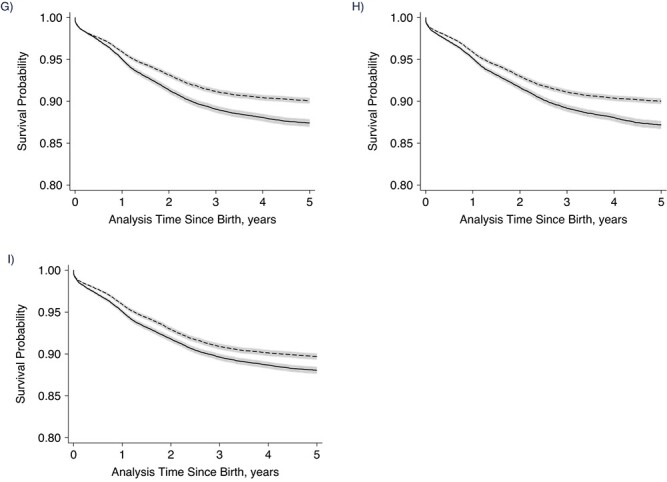
Unadjusted Kaplan-Meier estimates of child survival according to timing of exposure to reduced crop yields in the Nouna Health and Demographic Surveillance System, Burkina Faso, 1994–2016. Exposure was measured via the food crop productivity index (FCPI). A) Maternal FCPI exposure during the 12 months before the child’s conception; B) child’s gestational FCPI exposure; C) child’s FCPI exposure during the first 5.9 months after birth; D) child’s FCPI exposure at ages 6.0 months–1.9 years; E) child’s FCPI exposure at ages 2.0–4.9 years; F) child’s FCPI exposure during the first 1.9 years after birth; G) child’s FCPI exposure during the first 1,000 days after birth; H) child’s lifetime average FCPI exposure; I) child’s FCPI exposure during the first year after birth. The solid line shows the probability of survival in children exposed to FCPI < 100%; the dashed line shows the probability of survival in children exposed to FCPI ≥ 100%. Gray shaded areas show the 95% confidence intervals.

The unadjusted Kaplan-Meier plots suggested poorer survival for children exposed to a below–period-average FCPI in all time windows except exposure after the second birthday (Figure 2). The difference in survival rates in relation to the FCPI appears to have been greatest for exposure during 3 overlapping time windows: the child’s lifetime, the first 1,000 days from conception, and the first 1.9 years since birth.

**Figure Continues:**
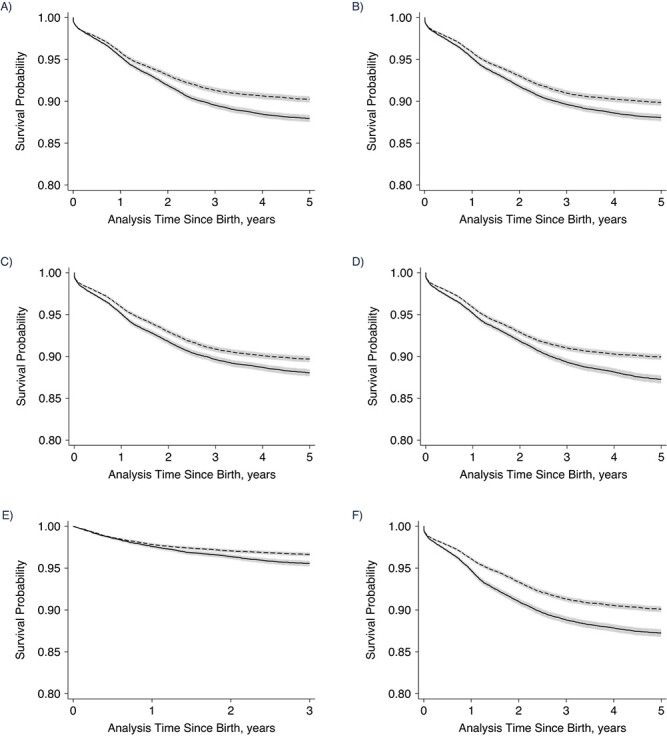


Results of Cox models with 1 exposure indicator fitted per model were largely in accord with the Kaplan-Meier plots (Table 3), with relatively large (and statistically significant) point estimates for the first 1,000 days from conception, the child’s lifetime, and the age 6.0 months–1.9 years time window and lower (and statistically nonsignificant) results for the preconceptional, gestational, age ≤5.9 months, and age 2.0–4.9 years windows. Sensitivity analyses based on the subset of villages that had been part of the HDSS since its inception showed similar results (Web Table 2).

**Table 3:** Survival to Age 4.9 Years in Relation to Individual Exposure to Reduced Food-Crop Yields (Cox Regression Analysis) Among Mothers and Children in the Nouna Health and Demographic Surveillance System, Burkina Faso, 1994–2016

		**Model 1** ^**a**^		**Model 2** ^**b**^		**Model 3** ^**c**^		
**FCPI Exposure Period**	**No. of** **Children**	**HR**	**95% CI**	**HR**	**95% CI**	**HR**	**95% CI**	**AIC** ^**d**^
Before conception	57,288	1.37	1.28, 1.47	1.08	1.00, 1.17	1.06	0.98, 1.14	113,728.7
First 1,000 days from conception	57,288	1.44	1.35, 1.55	1.13	1.04, 1.23	1.12	1.03, 1.22	113,723.3
Gestation	57,288	1.28	1.20, 1.37	1.06	0.99, 1.14	1.05	0.98, 1.14	113,728.6
Age ≤1.9 years	57,288	1.41	1.31, 1.51	1.12	1.03, 1.22	1.12	1.03, 1.22	113,868.9
Age ≤5.9 months	57,288	1.28	1.19, 1.37	1.05	0.97, 1.13	1.04	0.97, 1.12	113,729.4
Ages 6.0 months–1.9 years	57,288	1.34	1.26, 1.42	1.10	1.02, 1.18	1.10	1.03, 1.19	113,723.7
Ages 2.0–4.9 years^e^	42,624	1.21	1.06, 1.39	0.87	0.75, 1.02	0.88	0.75, 1.03	29,029.9
Child’s lifetime average	57,288	1.34	1.26, 1.42	1.10	1.02, 1.18	1.10	1.02, 1.18	113,723.9
First year after birth	57,288	1.27	1.20, 1.36	1.08	1.01, 1.16	1.08	1.01, 1.16	113,726.2

Abbreviations: AIC, Akaike information criterion; CI, confidence interval; FCPI, food crop productivity index; HR, hazard ratio.

^a^ Model 1 had random effects (shared frailty by village) and no adjustment for other variables.

^b^ In addition to model 1 adjustments, model 2 adjusted for the presence of an undernutrition treatment program (indicator of a step change in 2007) and time trend.

^c^ In addition to model 2 adjustments, model 3 adjusted for season of birth, sex, ethnicity, religion, mother’s and father’s ability to read, household’s wealth index, the presence of any household members involved in a nonagricultural occupation, level of village infrastructural development, and semirural versus rural residence.

^d^ AIC values are presented for the fully adjusted model (model 3).

^e^ To enable fitting of the FCPI exposure measure over 2.0–4.9 years of age, the analysis data set had to be restricted to the observations of those children who survived to and remained present in the Nouna Health and Demographic Surveillance System at 2 years of age.

Cox models that simultaneously included indices for more than 1 nonoverlapping exposure window showed comparatively large HRs for exposure in the window from 6.0 months to 1.9 years of age (Table 4 and Web Table 3). For example, the HR for 6.0 months–1.9 years was 1.15 (95% confidence interval (CI): 1.04, 1.26) for a 90th-to-10th percentile yield reduction when the model also included indices of the preconceptional, gestational, and first 5.9 months after birth windows. Evidence for the associations of child survival with preconceptional and gestational exposures was generally weak—HRs for the same level of crop yield reduction were 1.06 (95% CI: 0.97, 1.15) and 1.04 (95% CI: 0.93, 1.16), respectively. We found no evidence for an association with exposure during the first 5.9 months after birth.

**Table 4:** Survival to Age 4.9 Years in Relation to Multiple Simultaneously Fitted Indices of Exposure to Reduced Food-Crop Yields (Cox Regression Analysis) Among Mothers and Children in the Nouna Health and Demographic Surveillance System, Burkina Faso, 1994–2016

		**Model 1** ^**a**^		**Model 2** ^**b**^		**Model 3** ^**c**^	
**FCPI Exposure Period**	**No. of** **Children**	**HR**	**95% CI**	**HR**	**95% CI**	**HR**	**95% CI**
Models applied to full data set^d^	57,288						
Before conception		1.31	1.22, 1.41	1.08	1.00, 1.18	1.06	0.97, 1.15
Gestation		1.05	0.94, 1.16	1.03	0.92, 1.14	1.04	0.93, 1.16
Age ≤5.9 months		0.92	0.82, 1.04	0.93	0.82, 1.06	0.92	0.81, 1.04
Ages 6.0 months–1.9 years		1.34	1.23, 1.46	1.14	1.03, 1.25	1.15	1.04, 1.26
Models applied to restricted data set^e^	42,624						
Before conception		1.45	1.25, 1.67	1.16	0.98, 1.37	1.14	0.96, 1.34
Gestation		1.08	0.88, 1.31	0.99	0.81, 1.22	0.99	0.81, 1.22
Age ≤5.9 months		0.97	0.80, 1.18	0.92	0.76, 1.13	0.92	0.75, 1.12
Ages 6.0 months–1.9 years		1.23	0.93, 1.63	0.88	0.65, 1.20	0.88	0.65, 1.20
Ages 2.0–4.9 years		1.05	0.83, 1.34	0.97	0.75, 1.24	0.97	0.75, 1.24

Abbreviations: CI, confidence interval; FCPI, food crop productivity index; HR, hazard ratio.

^a^ Model 1 had random effects (shared frailty by village) and no adjustment for other variables.

^b^ In addition to model 1 adjustments, model 2 adjusted for the presence of an undernutrition treatment program (indicator of a step change in 2007) and time trend.

^c^ In addition to model 2 adjustments, model 3 adjusted for season of birth, sex, ethnicity, religion, mother’s and father’s ability to read, household’s wealth index, the presence of any household members involved in a nonagricultural occupation, level of village infrastructural development, and semirural versus rural residence.

^d^ Models were applied to the full data set, without adjustment for FCPI exposure at ages 2.0–4.9 years.

^e^ Models were applied to the restricted data set, with adjustment for FCPI exposure at ages 2.0–4.9 years. To enable simultaneous adjustment for FCPI exposure over 2.0–4.9 years of age, the analysis data set had to be restricted to the observations of those children who survived to and remained present in the Nouna Health and Demographic Surveillance System at 2 years of age.

When the analysis was restricted to children who survived to age 2 years, to enable adjustment for exposures experienced at 2.0–4.9 years of age, there was no association detected for any of these exposure windows. However, the power to detect such an association was limited by the smaller number of child observations eligible for these analyses (42,624 children vs. 57,288 children in other models).

The analyses stratified by wealth suggested a higher point estimate for the HR of child survival with FCPI exposure in the age window of 6.0 months–1.9 years in the poorest quartile of households (Table 5). However, the evidence for these associations was weak because of the small number of observations per stratum.

**Table 5:** Survival to Age 4.9 Years in Relation to Exposure to Reduced Food-Crop Yields (Food Crop Productivity Index) During the Age Period 6.0 Months–1.9 Years, According to Household Wealth, Among Children in the Nouna Health and Demographic Surveillance System, Burkina Faso, 1994–2016

		**Model 1** ^**a**^		**Model 2** ^**b**^		**Model 3** ^**c**^	
**Level of Household Wealth**	**No. of** **Children**	**HR**	**95% CI**	**HR**	**95% CI**	**HR**	**95% CI**
Level 1 (poorest)	7,950	1.40	1.21, 1.63	1.18	0.99, 1.41	1.17	0.98, 1.40
Level 2	8,448	1.21	1.04, 1.40	1.12	0.94, 1.33	1.11	0.93, 1.33
Level 3	8,012	1.24	1.06, 1.45	0.99	0.82, 1.20	0.97	0.80, 1.17
Level 4 (wealthiest)	3,469	1.22	0.99, 1.52	1.15	0.89, 1.49	1.15	0.89, 1.49
Unclassified	11,091	1.35	1.17, 1.56	1.16	0.98, 1.38	1.17	0.99, 1.40

Abbreviations: CI, confidence interval; FCPI, food crop productivity index; HR, hazard ratio.

^a^ Model 1 had random effects (shared frailty by village) and no adjustment for other variables.

^b^ In addition to model 1 adjustments, model 2 adjusted for the presence of an undernutrition treatment program (indicator of a step change in 2007) and time trend.

^c^ In addition to model 2 adjustments, model 3 adjusted for season of birth, sex, ethnicity, religion, mother’s and father’s ability to read, household’s wealth index, the presence of any household members involved in a nonagricultural occupation, level of village infrastructural development, and semirural versus rural residence.

## DISCUSSION

This study contributes new empirical evidence on the understudied question of the timing of children’s vulnerability to early-life exposures to interannual crop yield reduction in a subsistence farming population in sub-Saharan Africa. To our knowledge, it is the first study providing a detailed analysis of children’s vulnerability to crop yield reductions according to the key developmental stages in early life.

**Figure 3:**
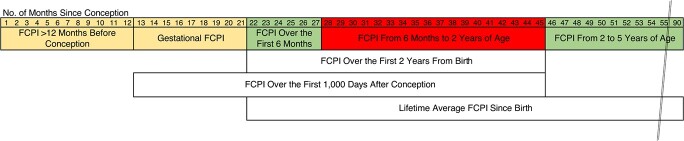
Summary of findings on the associations of child survival with annual crop yield reductions during different windows of exposure, Nouna Health and Demographic Surveillance System, Burkina Faso, 1994–2016. Red represents a strong association, yellow represents a borderline/weak association, and green represents no significant association. FCPI, food crop productivity index.

Although it is difficult to provide precise interpretation of the relative importance of different time windows, the evidence appears strongest for low FCPI’s being important during the period from age 6.0 months to age 1.9 years (Figure 3). The evidence was generally weaker for preconception and gestational exposures, for age ≤5.9 months, and for the period from age 2 years onwards.

In our study subjects, the leading immediate causes of death in the age group 6.0 months–1.9 years were malaria (60%), diarrhea and other gastrointestinal infections (11%), respiratory infections (8%), and meningitis (3%). Undernutrition is underreported as a cause of death, since it underlies other causes and is rarely noted by physicians among the causes of death (29). It is estimated that globally undernutrition underlies 45% of deaths in children under 5 years of age (30). In Burkina Faso, cereal crops are essential for preparing porridges that are commonly introduced into children’s diets as complementary food at the age of 6 months alongside breastfeeding and continued until 2 years of age (31). Vulnerability to cereal-crop yield reductions in the window from 6.0 months to 1.9 years of age could be explained by the nutritional dependence on cereal-crop products in this age group, possibly further amplified by often late introduction of complementary feeding in this setting (31). Reduced crop yields may lead to suboptimal food intake and limited means of accessing health care, thus making children in this age group more vulnerable to the immediate causes of death in this age group. Our findings of weaker evidence in relation to cereal-crop yield reductions in utero and during the first 5.9 months after birth could be related to physiological mechanisms that protect the fetus nutritionally—for example, placental phenotype alteration in circumstances of maternal nutrient restriction that occurs to maintain normal fetal growth, and breastfeeding (32). Similarly, the protective effect of immunization and conditionality of survival to 1.9 years of age (“survival of the fittest”) could to some degree explain our finding of no evidence for an association with exposures incurred between 2.0 and 4.9 years of age.

We are aware of only 1 prior study that examined child mortality in relation to *both* prenatal and early postnatal exposure to annual crop yield variation, a 2014 study by Johnson and Brown (16). It partly supports our findings, with observed associations between child mortality and NDVI in the first year of life in dry settings (Burkina Faso in 2003, Mali in 2001) but not in other settings (Benin in 2001, Guinea in 2005, Mali in 2006) (16); but whereas we found weak evidence for prenatal exposure, the smaller (less powerful) and cross-sectional Johnson and Brown study found no association (16). Further studies examined crop yield variation proxies in the pre- and early postnatal periods only in relation to child stunting. Stunting can be experienced as a result of restricted food intake in early life and the periconceptional period and is strongly associated with subsequent risk of mortality (8, 14, 23). It may therefore partly mediate the association between child mortality and early-life exposure to crop yield fluctuations. The results of these studies are not entirely consistent. The majority suggested evidence for associations with both pre- and postnatal (first to second year after birth) exposures to crop yield reductions and their proxy indicators, NDVI and rainfall during crop growth (15, 17, 18). Only 1 study found no evidence of association of stunting and prenatal exposures with NDVI and mixed evidence in relation to exposure during the period from birth to 2 years of age (16).

The differences in findings across studies could be due to differences in social, economic, and political circumstances, which influence access to food, food prices, and levels of support and health care, as well as the extent of the observed crop yield/NDVI variation (16). Mechanisms may also differ across settings and by timing—for example, an impact operating through decreased household food availability versus income and resource availability for health-care needs versus weather-related changes in vectorborne disease risk. An adequate micronutrient intake may be more important during the perinatal period and in utero, while protein-energy intake may be more important in the postnatal stages (7, 8). Furthermore, NDVI does not fully capture crop yield variations, since it detects only the intensity of vegetation cover, not the extent of grain formation, which can be affected by rainfall shocks at specific stages of crop growth without a visible impact on vegetative parts of the plant (33). More advanced methods of microyield monitoring using satellite imagery at different stages of crop growth offer far more accurate yield assessment (34). Equally, the effect of spatial variability in the NDVI—as captured, for example, by Johnson and Brown (16)—may not be directly comparable with the effects of interannual variability in crop yield examined in our analyses.

Our findings of vulnerability to early-life reductions in crop yields in our study population, and potentially in similar populations, are of particular concern given the projections of further crop yield reductions and increased frequency and intensity of droughts in West Africa with future climate change (35). In earlier work, we established that 72% of interannual variation in crop yields is related to adverse weather patterns during the crop-growing season (36). Based on our previously identified association of child survival with crop yield variations in the year of birth (20), we estimated that changes in the frequency of adverse weather patterns and their impact on crop yield appreciably increased child mortality in Nouna even under the aspirational target of maintaining a global average temperature increase below 1.5^ο^C (36). Our current analyses of separate windows of child vulnerability to crop yield reductions showed an even higher mortality HR in relation to crop yield reductions in the window of age 6.0 months–1.9 years (20), which may have a bearing on the impact of climate change on child mortality. There is a need and opportunity to design more effective adaptation strategies to crop yield reductions and the processes that lead to child vulnerability in the 6.0 months–1.9 years time window—for example, strengthening the resilience of healthy complementary feeding practices in times of food insecurity. If further evidence from other settings supports our findings, regular monitoring efforts would need to be prioritized in this portion of the population in Burkina Faso and the wider region to protect against potential increases in the effects of low crop productivity on child health. More resilient agricultural systems and improved management practices are also essential.

As in any observational study, we cannot exclude the possibility of residual confounding. However, we adjusted our analyses for all known time-varying confounders, including a linear time trend and the introduction of an undernutrition treatment program (20). In contrast to our earlier work (20), the current analyses explored and incorporated further adjustments for socioeconomic differences (household wealth, involvement in nonagricultural employment, village infrastructural development). Because the household wealth data were available only from the year 2009, we had to assume the wealth values from that year for the entire study period. Yet, the effect of wealth on child mortality was not the main focus of our analyses. The study design compared the entire population against itself in one year versus another, and, as our results demonstrate, the effect of adjusting our analyses for individual and household characteristics was minimal. Therefore, the time-invariant nature of the wealth index is unlikely to have had a notable bearing on our results. Our identified associations remained statistically significant regardless of these adjustments. Our analyses did not allow us to distinguish the extent to which the association of food crop yield variations with child mortality was mediated by the child’s own cereal-crop consumption versus cereal-crop purchases or sales; this remains an area for future research. However, studies carried out in the area suggest that about 90% of what households eat has been grown and harvested in their own fields (37). Finally, the extent to which we were able to compare the importance of specific time windows of children’s exposure to crop yield variations was limited by the nature of the local agricultural calendar, with only 1 crop cultivation season per year. As a result, the exposure indices often overlapped by at least 1 agricultural year, and hence 1 common yield value. For example, a child born on December 20, 2018, would have been exposed in utero to a weighted average of yield values of the harvests of September 2017 and 2018 and exposed in the first 2 years of life to the weighted average of the yield values of the harvests of September 2018, 2019, and 2020. Hence, the 2 exposure values would to some extent be interdependent, as both would incorporate the yield value of the same harvest of September 2018. Similar analyses conducted in settings with multiple crop seasons per calendar year would provide greater variability across the exposure indices reflecting different time periods of early-life development, and might therefore provide a clearer comparison of the differential effects of exposures in one time window versus another. In future research, investigators could attempt to develop and use estimates of crop yield variability at a higher spatial resolution than the provincial level to capture spatiotemporal variability in crop yields across the study population, capturing such localized disruptions to crop production as flooding. Other topics to explore in future work include analyses of how access to health care mediates the association between annual crop yield variation and child mortality.

In conclusion, in the Nouna area of Burkina Faso, child health appears to be particularly vulnerable to cereal-crop yield reductions during the period from 6.0 months to 1.9 years of age—the period of complementary feeding, often using cereal porridges alongside breastfeeding. This finding is particularly important in the context of the projected reductions and increased unpredictability of crop yields due to increased weather variability from climate change in West Africa. It suggests opportunities for improving the effectiveness and efficiency of climate change adaptation policies for nutrition and health, as well as for other nutritional interventions and support in our study area and potentially similar areas.

## Supplementary Material

Web_Material_kwad068
