# Supplementary material for: Child Survival and Annual Crop Yield Reductions in Rural Burkina Faso: Critical Windows of Vulnerability Around Early-Life Development
Source: Am J Epidemiol. 2023 Apr 28;192(7):1116–27. doi: 10.1093/aje/kwad068 (PMC10326605; doi:10.1093/aje/kwad068)
Supplement: Web_Material_kwad068 [file web_material_kwad068.pdf]

WEB MATERIAL

Child Survival and Annual Crop Yield Reductions in Rural Burkina Faso: Critical Windows of Vulnerability Around Early-Life Development

Kristine Belesova, Antonio Gasparrini, Paul Wilkinson, Ali Sié, and Rainer Sauerborn

Table of Contents

Web Table 1. Correlation coefficients of all the examined FCPI exposure metrics by timing of exposure…………….2

Web Figure 1. Time series of the Food Crop Productivity Index (FCPI) (A) and annual yield of each individual crop comprising the FCPI (B) in Kossi Province, Burkina Faso, 1992–2016………………..……………………………….2

Web Table 2. Results of the sensitivity analyses replicating the Cox regression analyses when the data set is restricted to the 46 villages that were part of the Nouna HDSS since 1994: child survival to 4.9 years of age in relation to individual food crop yield exposure indices in Nouna HDSS, Burkina Faso, 1994–2016………………………………3

Web Table 3. Results of Cox regression analysis: child survival to 4.9 years of age in relation to multiple simultaneously fitted exposure indices in Nouna HDSS, Burkina Faso, 1994–2016……………………………………4

Web Table 2. Correlation coefficients of all the examined FCPI exposure metrics by timing of exposure

	**Before Conception**	**Gestation**	**≤5.9 Months**	**6.0 Months–1.9 Years**	**2.0–4.9 Years**	**Year of Birth**	**≤1.9 Years**	**First 1,000 Days**	**Lifetime Average**
Before conception	1.00								
Gestation	0.44	1.00							
≤5.9 months	0.28	0.69	1.00						
6.0 months–1.9 years	0.10	0.22	0.38	1.00					
2.0–4.9 years	0.06	-0.02	0.18	0.80	1.00				
Year of birth	0.31	0.85	0.87	0.27	-0.01	1.00			
≤1.9 years	0.09	0.40	0.55	0.75	0.30	0.54	1.00		
First 1,000 days	0.24	0.69	0.69	0.68	0.23	0.74	0.94	1.00	
Lifetime average	0.12	0.28	0.47	0.98	0.74	0.37	0.83	0.76	1.00

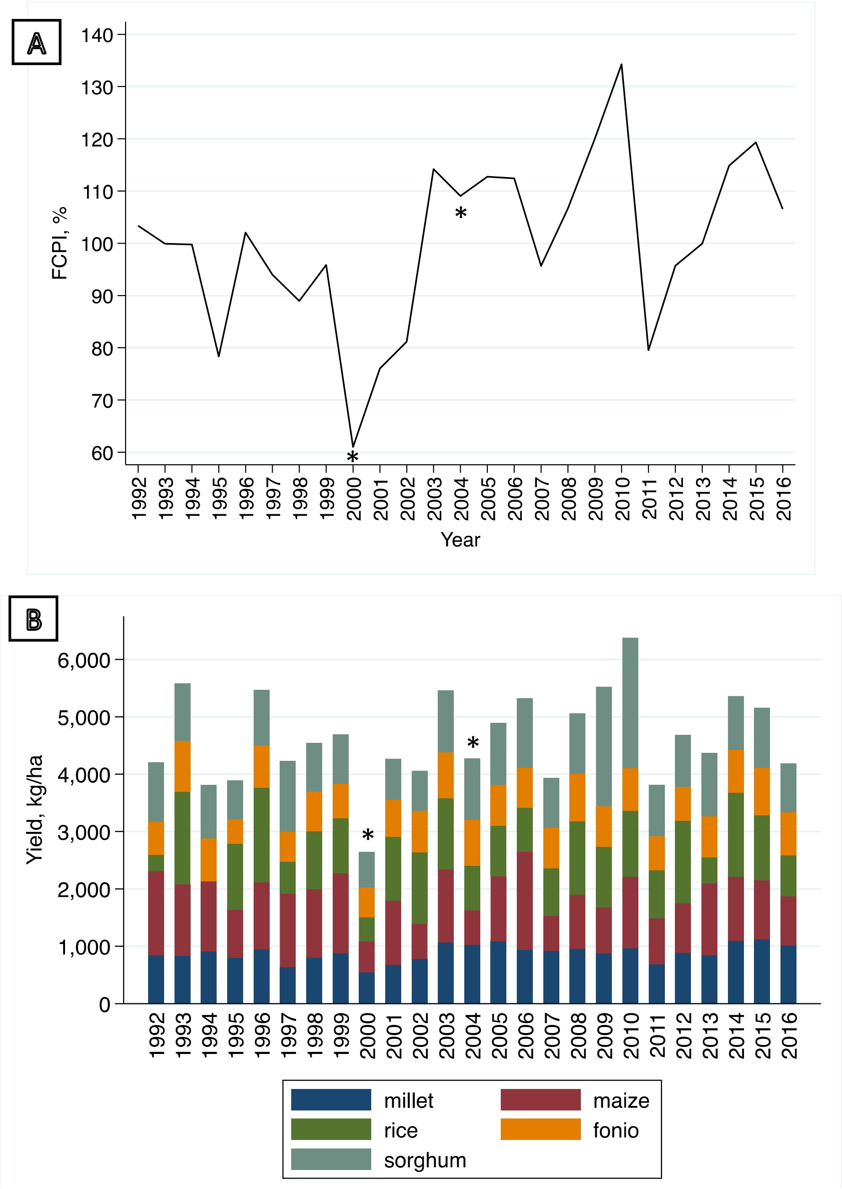

Web Figure 2. Time series of the food crop productivity index (FCPI) (A) and annual yield of each individual crop comprising the FCPI (B) in Kossi Province, Burkina Faso, 1992–2016.

** The HDSS population was expanded in the years 2000 and 2004.*

Web Table 2. Results of the sensitivity analyses replicating the Cox regression analyses when the data set is restricted to the 46 villages that were part of the Nouna HDSS since 1994: child survival to 4.9 years of age in relation to individual food crop yield exposure indices in Nouna HDSS, Burkina Faso, 1994–2016

FCPI Exposure	No. of Children	Model 1[a]		Model 2[b]		Model 3[c]	
		HR	95% CI	HR	95% CI	HR	95% CI
Before conception	38,970	1.34	1.23, 1.45	0.93	0.98, 1.17	1.04	0.96, 1.14
First 1,000 days	38,970	1.41	1.30, 1.53	1.15	1.04, 1.27	1.15	1.04, 1.27
Gestation	38,970	1.26	1.17, 1.36	1.07	0.98,1.16	1.06	0.97, 1.16
≤1.9 years	38,970	1.38	1.27, 1.49	1.15	1.05, 1.26	1.15	1.04, 1.26
≤5.9 months	38,970	1.26	1.16,1.36	1.07	0.98, 1.17	1.06	0.97, 1.16
6.0 months–1.9 years	38,970	1.30	1.21, 1.40	1.11	1.02, 1.21	1.11	1.02, 1.21
2.0–4.9 years	29,138[d]	1.22	1.05, 1.42	0.84	0.70,1.01	0.84	0.70, 1.01
Lifetime average	38,970	1.30	1.21, 1.40	1.11	1.02, 1.21	1.11	1.02, 1.21
Year of birth	38,970	1.26	1.17, 1.35	1.10	1.01, 1.19	1.09	1.01, 1.19

Abbreviations: CI, confidence interval; HR, hazard ratio; FCPI, food crop productivity index.

[a] Model 1 with random effects (shared frailty by village), unadjusted for other variables.

[b] Model 2 additionally to Model 1 adjustments, adjusted for the presence of undernutrition treatment program (indicator of step change in 2007), time trend.

[c] Model 3 additionally to Model 2 adjustments, adjusted for season of birth, sex, ethnicity, religion, mother's and father's ability to read, household's wealth index, presence of any members in the household involved in a non-agricultural occupation, level of village infrastructural development, and semi-rural *vs* rural residence.

[d] To enable fitting the FCPI exposure over 2.0–4.9 years of age, the analyses data set had to be restricted to the observations of these children who survived to and remained present in Nouna HDSS at 1.9 years of age.

Web Table 3. Results of Cox regression analysis: child survival to 4.9 years of age in relation to multiple simultaneously fitted exposure indices in Nouna HDSS, Burkina Faso, 1994–2016

FCPI Exposure Timing	No. of Children	Model 1[a]		Model 2[b]		Model 3[c]	
		HR	95% CI	HR	95% CI	HR	95% CI
Simultaneously fitted:							
Gestation	57,288	1.11	1.03, 1.20	1.02	0.94, 1.10	1.01	0.93, 1.10
≤1.9 years		1.33	1.23, 1.44	1.11	1.02, 1.22	1.12	1.02, 1.22
Simultaneously fitted:							
Gestation	57,288	1.19	1.08, 1.31	1.07	0.96, 1.18	1.06	0.96, 1.18
≤5.9 months		0.90	0.80, 1.02	0.93	0.82, 1.05	0.91	0.81, 1.04
6.0 months–1.9 years		1.33	1.22, 1.45	1.12	1.02, 1.24	1.14	1.04, 1.25
Simultaneously fitted:							
Gestation	57,288	1.13	1.05, 1.22	1.03	0.95, 1.11	1.02	0.94, 1.10
Lifetime average		1.27	1.18, 1.36	1.09	1.00, 1.18	1.09	1.01, 1.18
Simultaneously fitted:							
Before conception	57,288	1.33	1.23, 1.44	1.09	1.01, 1.19	1.07	0.98, 1.16
Gestation		0.97	0.89, 1.06	0.98	0.90, 1.07	0.98	0.90, 1.07
≤1.9 years		1.37	1.27, 1.49	1.14	1.04, 1.25	1.13	1.04, 1.24
Simultaneously fitted:							
Before conception	57,288	1.31	1.22, 1.41	1.08	1.00, 1.18	1.06	0.97, 1.15
Gestation		1.05	0.94, 1.16	1.03	0.92, 1.14	1.04	0.93, 1.16
≤5.9 months		0.92	0.82, 1.04	0.93	0.82, 1.06	0.92	0.81, 1.04
6.0 months–1.9 years		1.34	1.23, 1.46	1.14	1.03, 1.25	1.15	1.04, 1.26
Simultaneously fitted:							
Before conception	57,288	1.15	1.06, 1.24	1.08	1.00, 1.18	1.06	0.97, 1.15
Gestation		0.98	0.90, 1.07	0.99	0.91, 1.08	0.99	0.91, 1.08
Lifetime average		1.06	0.98, 1.15	1.10	1.02, 1.19	1.10	1.02, 1.20
Simultaneously fitted:							
Before conception	57,288	1.28	1.19, 1.37	1.07	0.99, 1.15	1.04	0.97, 1.13
First 1,000 days		1.36	1.26, 1.46	1.12	1.03, 1.22	1.12	1.03, 1.22
Simultaneously fitted:							
First 1,000 days	42,624	1.23	1.05, 1.43	0.80	0.66, 0.961	0.79	0.65, 0.96
2.0–4.9 years		1.16	1.01, 1.34	0.87	0.75, 1.02	0.88	0.75, 1.02
Simultaneously fitted:							
Gestation	42,624	1.29	1.08, 1.54	1.05	0.87, 1.27	1.05	0.86, 1.27
≤5.9 months		0.96	0.79, 1.16	0.92	0.75, 1.12	0.91	0.74, 1.11
6.0 months–1.9 years		1.13	0.85, 1.49	0.83	0.62, 1.12	0.84	0.62, 1.13
2.0–4.9 years		1.15	0.90, 1.45	0.99	0.77, 1.27	0.99	0.77, 1.27
Simultaneously fitted:							
Before conception	42,624	1.45	1.25, 1.67	1.16	0.98, 1.37	1.14	0.96, 1.34
Gestation		1.08	0.88, 1.31	0.99	0.81, 1.22	0.99	0.81, 1.22
≤5.9 months		0.97	0.80, 1.18	0.92	0.76, 1.13	0.92	0.75, 1.12
6.0 months–1.9 years		1.23	0.93, 1.63	0.88	0.65, 1.20	0.88	0.65, 1.20
2.0–4.9 years		1.05	0.83, 1.34	0.97	0.75, 1.24	0.97	0.75, 1.24

Abbreviations: CI, confidence interval; HR, Hazard ratio; FCPI, food crop productivity index.
[a] Model 1 with random effects (shared frailty by village), unadjusted for other variables.
[b] Model 2 additionally to Model 1 adjustments, adjusted for the presence of undernutrition treatment program (indicator of step change in 2007), time trend.
[c] Model 3 additionally to Model 2 adjustments, adjusted for season of birth, sex, ethnicity, religion, mother's and father's ability to read, household's wealth index, presence of any members in the household involved in a non-agricultural occupation, level of village infrastructural development, and semi-rural *vs* rural residence.
